# Supplementary material for: Improving work detection by segmentation heuristics pre-training on factory operations video
Source: PLoS One. 2022 Jun 7;17(6):e0267457. doi: 10.1371/journal.pone.0267457 (PMC9173629; doi:10.1371/journal.pone.0267457)
Supplement: Attachment — Submitted filename: Response to Reviwers_final_publish.docx [file pone.0267457.s001.docx]

Manuscript: PONE-D-21-26196

Response to Reviewers

Editor

1. If applicable, we recommend that you deposit your laboratory protocols in protocols.io to enhance the reproducibility of your results. Protocols.io assigns your protocol its own identifier (DOI) so that it can be cited independently in the future. For instructions see: https://journals.plos.org/plosone/s/submission-guidelines#loc-laboratory-protocols. Additionally, PLOS ONE offers an option for publishing peer-reviewed Lab Protocol articles, which describe protocols hosted on protocols.io. Read more information on sharing protocols at https://plos.org/protocols?utm_medium=editorial-email&utm_source=authorletters&utm_campaign=protocols.

Response: We did not use “Protocols.io” due to the time limitation, since the source code was newly published and the flow was defined in detail. We would like to consider it in the future works.

Reviewer 1

**1.**	Paper aims to present an improvement (from performance perspective) of a classic CNN-LSTM model for computing spatio-temporal information. Introduction promotes "a method for considering heuristics in a model that uses an image segmentation task as a pre-training method" as a key novelty of the paper in addition to the dataset provided at Google's server. I'm not so sure that this method is unique, as similar approaches have been done before in a different context. This requires a clarification. Authors must provide an in-depth mathematical apparatus (missing completely, only generic statements were made) and full UMLs of the implementation, including the architecture along with necessary hyper parameters of the network. Pseudo codes provided are too shallow, failing to showcase what exactly is different from other approaches on computing spatio-temporal information. Must be expanded.

Response: We thank the reviewer for the recommendation. We have added the following sentences.

Uniqueness: we added a sentence to the introduction section.

“There has been no research on the application of such a method to work videos in factories. In particular, the video proposed in this study has completely new characteristics, as described in Table 2, and it would be of great academic value to confirm the effectiveness of heuristic pre-training on this video.”

Implementation details: we added or fixed figures and sentences to the method section.

Figure2(fixed), Figure3(added)

“The CNN-Module used in our experiment is shown in Figure 3. As the CNN module, we adopted the Encoder of DeepLab v3+ [116] that we utilize for pretraining, as described in the following section. Since the output of DeepLab v3+ Encoder is a feature map, we added Vectorizer right after Encoder to convert it into a vector”

We published the CNN-LSTM code and added the following sentence to the introduction.

“The code is available at https://github.com/ShotaroKataoka/work_detection_factory_pretrain.”

2.	Provide loss curves (prove that your solution is not over-fitting) and tsne.

Response: We thank the reviewer for the comment. We have added the following sentences and figures.

Loss curve: we added a sentence and a figure to the experiment section.

Figure5(added)

“Figure 5 shows a loss curve of our factory-segmentation-pre-trained CNN-LSTM. For prediction, we used the weights of the epochs with the highest class accuracy in the validation set. In this experiment, the best validation class accuracy was achieved at 9 epochs (training took 21 minutes), so we used the weights at 9 epochs for the experiment. In the loss curve, the validation loss and training loss both show a decreasing trend, and there is no significant overfitting. Although there is some gap between these two, it is considered to be within the acceptable range for learning progress.”

t-SNE: we added a sentence and a figure to the discussion section.

Figure6(added)

“Figure 6 shows the output feature vectors of factory segmentation pre-trained CNN-LSTM dimensionally reduced using t-SNE and mapped onto a two-dimensional graph. The 64-dimensional feature vector, which is the output of the LSTM prior to the FC+Softmax layer in Figure 2, was compressed into a 2-dimensional vector by t-SNE. The parameters of t-SNE were set to perplexity = 30 and n_iter = 1000. t-SNE dimensionality reduction was performed on the entire test set, from which 12,000 samples were randomly sampled so that all classes were evenly distributed, and a scatter plot was created.”

3.	Add explanation to figure 4. What each class means.

Response: We thank the reviewer for the recommendation. We have fixed figure4 and its explanation.

- Figure 4 has been changed to Table 10.

“Table 10 shows the confusion matrix of result of our CNN-LSTM model of test set. The rows show the classes of the groundtruths (GT). The columns show the classes predicted by the model (PR). The value of each element is a count of the pairs of the GT and the PR. The model predicted once per second.”

“Table 10: Confusion matrix of our model’s result (test set). The rows show the classes of the groundtruths (GT). The columns show the classes predicted by the model (PR). The value of each element is a count of the pairs of the GT and the PR. The diagonal line is the true positive and is expressed in a bold font. The class numbers in the table correspond to the class IDs in Table 4.”

4. Experimental part does not detail the equipment HW and SW infrastructure used.

Response: We thank the reviewer for the comment. We have added the Figure 5 and a sentence to the experiment section.

“Table 5 shows the equipment hardware and software infrastructure details of our experiment. Computational resource of AI Bridging Cloud Infrastructure (ABCI) provided by National Institute of Advanced Industrial Science and Technology (AIST) was used.”

5.	Performance part (this method was supposedly much faster than accurate vs others) was not covered sufficiently.

Response: We thank the reviewer for the comment. We have added the following sentence to the experiment section.

“training took 21 minutes”

5. Literature review is organized, but contains too few references. There has been a lot done in this area. Estimate around 20+ papers per section easily

Response: We thank the reviewer for the comment. As the reviewer recommended, we have added total 76 references and the detailed explanation written in red in the manuscripts. And we have also updated the “Table 2; Comparison of video datasets”.

6. Image (video) data is available on the google drive. Yet the aggregated raw results were not provided.

Response: We thank the reviewer for the comment. We have uploaded raw results to the google drive and added the following sentence to the introduction section.

“The raw result is available along with the dataset described above.”

7.	Please back up this statement "manufacturing industry, the use of local computers to make inferences is often required for practicality and confidentiality reasons, necessitating a low computational cost, and so the latter, a lightweight model, needs to have improved performance". Computing costs are negligible compared to manufacturing costs and equipment.

Response: Thank you for your comment. It is true that the cost of a computer may be small compared to the cost of equipment. However, this system is intended for small and medium-sized companies. However, this system is designed for small and medium-sized enterprises (SMEs), which do not have ample funds and cannot easily afford to purchase computers. Therefore, it is important to have a simple model that does not require a lot of machine power.

8. Paper focuses on analyzing workers (live human subjects) yet provides no ethical permit.

Response: We thank the reviewer for this comment. As described in the paper, the dataset videos were filmed using a fixed camera for three days at a specific work site in a printing factory (Echigo Fudagami Inc : Uenoyama 1-2-8, Ojiya, Niigata, Japan.), with a data size of 10 h per day, i.e., 30 h for the entire dataset. The data were collected after prior explanation to the company (Echigo Fudagami Inc) that the data would be used for research purposes and verbal consent was obtained from the company manager and the subject workers. Permission to conduct the research was obtained from the company manager and subject workers. Our university's ethics committee confirmed that ethical approval was waived. And no third-party ethical oversight was provided.

Reviewer 2

1. Introduction – a very good research question and a significant problem of working time analysis. However please express the area of recognition of employee activities in the production process. So please extend your introduction this area and add the sources: https://doi.org/10.3390/sym11091151; https://www.mdpi.com/2071-1050/13/10/5495.

Response: Thank you for your detailed response and valuable insights. We add the following sentence written in red into “Introduction” and 17 references included “https://www.mdpi.com/2071-1050/13/10/5495”.

“Recognition of employee activities in the production process is gaining attention \cite{Bell, E.:2020}-\cite{Graessley, S. :2019}. Among them, employee behavior recognition in the manufacturing industry is attracting increasing attention from both industry and academia \cite{Meyers, T. D.:2019}. The importance of real-time process monitoring and analysis of employee behavior based on various sensors and other devices is increasing from both industry and academia nowadays.\cite{White, T.:2020} - \cite{Davis, R.:2020}”

2. Related work – the authors assume that background is the same throughout the video. Is the environment at the adopted position really not changing? And if so, how can this problem be solved?

Response:

We thank the reviewer for the important question. Our target is a production line with many routine tasks. In this type of line, the background rarely changes, such as when the machines change. On the other hand, when the background changes, it is when the machine layout of the factory changes drastically. In such a factory where the layout changes, the work procedures and contents also change, so they are not the subject of the work analysis in this research. We will address this issue in the future.

3. In the video data set – the authors state, that the length of time to perform the same work consecutively varies considerably, ranging from a few seconds to tens of minutes, and tasks depend on one other over a very long period. Is the same activity performed by the same employee then? Are there no time standards for individual activities? Are the steps always performed in the same sequence?

Response:

We thank the reviewer for the question. In many companies, there are no individual time standards, so they have to be identified using this kind of method. Although work procedures may change, they are included in the scope of work process analysis, and this research is able to handle them.

4. Please add an explanation why DeepLab v3 was chosen as a segmentation model for pre-training - description: "it is a simple encoder – decoder model consisting of a single stage" is not sufficient.

Response: We thank the reviewer for the valuable comment. Deep Lab V3 is one of the best performers in the field of segmentation. For example, according to the ranking of https://paperswithcode.com/, it ranks 2nd in Pascal 2012 and 1st in Anatomical Tracings of Lesions After Stroke (ATLAS). In addition, it is widely used in practical applications and has stable operation.

5. How the findings can be exploited by future similar works?

Response: As described in the paper, unlike the conventional image recognition task, the analysis of manufacturing tasks is difficult because the work time varies greatly for each task. Since this research has solved this problem, we believe that we have achieved a significant result in that we have developed a standard model for future work analysis in manufacturing and a dataset that can be used for pre-training. This research can be used to analyze various tasks in the manufacturing industry, including the detection of abnormalities in work, and will contribute to the diversification of work analysis tasks.

6. Please add the limitations of the work.

Response: We thank the reviewer for the important comment. Our proposed method cannot be used when the factory layout changes drastically. If another task is added, relearning is required.
