# Supplementary material for: Improving work detection by segmentation heuristics pre-training on factory operations video
Source: PLoS One. 2022 Jun 7;17(6):e0267457. doi: 10.1371/journal.pone.0267457 (PMC9173629; doi:10.1371/journal.pone.0267457)
Supplement: Attachment — Submitted filename: Response to Reviwers_revision_3_2_publish.docx [file pone.0267457.s002.docx]

Manuscript: PONE-D-21-26196

Response to Reviewers

We have corrected the points pointed out by the reviewers. The red letters are the corrections made in the previous revision, and the blue letters are the corrections made in this revision. Underlines are grammatical corrections in the previous revision.

Reviewer 1

**1.**	English language still requires a rework.

Response:

We thank the reviewer for the recommendation. We have made some grammatical corrections to our manuscript. We have underlined the corrections.

2.　Statistical analysis still requires more effort to prove the validity.

Response:

We thank the reviewer for the recommendation. We performed a Welch's t-test on the results. We have added the following sentences to the experiment section.

“In addition, we performed a Welch’s t-test to confirm the significance of the difference between the output of each method. As a specific procedure, we randomly divided the dataset into 100 parts, calculated the F1 score for each class in each part, and performed Welch's t-test on each class.

Consequently, the null hypothesis on all classes F1 score can be rejected at the 1\% significance level for the difference between baseline and ours.”

Reviewer 2

1. Why were 17 new literature references added and indicated in the recommendations not added?

Response:

We are very sorry about it. The number of citations has been reduced by mistake. We have added one reference that was not cited previously, and one more citation ([13],[14]). Therefore, we have added 18 citations from the first edition.

[13] Andronie, M., Lăzăroiu, G., Ștefănescu, R., Uță, C., & Dijmărescu, I. (2021). Sustainable, Smart, and Sensing Technologies for Cyber-Physical Manufacturing Systems: A Systematic Literature Review. Sustainability, 13(10), 5495.

[14] Patalas-Maliszewska, J., & Halikowski, D. (2019). A model for generating workplace procedures using a CNN-SVM architecture. Symmetry, 11(9), 1151.

The text has been changed to the following sentence.

“Recognition of employee activities in the production process is gaining 2 attention [1] [2] [3] [4] [5] [6] [7] [8] [9] [10] [11] [12]. Among them, employee behavior 3 recognition in the manufacturing industry is attracting increasing attention from both 4 industry and academia [13]. For example, to improve management and effectiveness of 5 employees ’learning processes, Patalas-Maliszewska et.al [14] used CNN-SVM to 6 extract features from video material concerning each work activity, and comparison 7 with the features of the instruction picture. The importance of real-time process 8 monitoring and analysis of employee behavior based on various sensors and other 9 devices has been increasing from both industry and academia [15] [16] [17] [18].”

2. Moreover, the most important problem that has not been explained in the article is the incorrect assumption that "In many companies, there are no individual time standards, so they have to be identified using this kind of method". In the production process, each workstation has a different duration of activity. You stated, that "Although work procedures may change, they are included in the scope of work process analysis". So you can use the method without any requirements? This aspect requires a thorough explanation. Also, my suggestion to present the limitations of the work was as also explained laconic.

Response:

We thank the reviewer for the recommendation. In our previous reply, we misinterpreted your question and gave a wrong answer.

Each individual task has its own time standards. Throughout the dataset, the worker is the same, but there seems to be a time variance of a few minutes to a few tens of minutes when the work procedures are different. An explanation of this has been added to the manuscript with an example.

Also, as you pointed out, the description of limitations in the paper was insufficient. We have added the following sentences to the limitations sub-section in the discussion section.

“In this dataset, the same person performs works throughout the entire dataset. Since our method includes abstraction of the body parts of the worker by segmentation, it is considered to be robust to changes in the workers. However, the method may not be able to infer correctly when the procedures and motions of the workers change significantly due to differences in the workers. In this case, we need to add new training data.

There were cases where the working time for the same task varied from several minutes to several tens of minutes. This is thought to be because the work procedures may differ even for the same work. For example, "Product check" work is performed continuously for several tens of minutes, but it may be interrupted irregularly when defective products are found, so the work may last from a few seconds to several minutes. Since the training data contains multiple work order patterns even for the same task, the model can predict when the work order changes to some extent. However, if the order of the tasks is highly irregular, or if the tasks are completely new and unknown, the inference may not be correct. In such cases, it is necessary to add training data or review the contents of the work labels.

Our method is robust to changes in background information that is irrelevant to the task by using the segmentation of critical elements. However, since the method assumes a fixed camera, the trained model cannot be used as-is when the layout of the machines and the work environment related to the work changes. In these cases, it is considered necessary to re-collect the training data.”
