# Supplementary material for: Improving work detection by segmentation heuristics pre-training on factory operations video
Source: PLoS One. 2022 Jun 7;17(6):e0267457. doi: 10.1371/journal.pone.0267457 (PMC9173629; doi:10.1371/journal.pone.0267457)
Supplement: Attachment — Submitted filename: Response to Reviwers_revision_4_1.docx [file pone.0267457.s003.docx]

Manuscript: PONE-D-21-26196

Response to Reviewers

We have corrected the points pointed out by the reviewers. The magenta letters are the corrections made in this revision.

**Editor**

There was a mistake in the program that calculates the scores, so it was corrected. As a result, each score is slightly different from the previous version, but the effect is slight and makes no difference to the final conclusions. We have released an executable score calculation program to keep the calculation process open. Also, in the previous versions, the maximum increase in class F1 was incorrectly listed where the increase in macro F1 should have been listed. We have corrected this. The score has been incorrect, but the discussion was originally based on the macro F1, so the conclusion and discussion remain unchanged.

**Reviewer 1**

**1.	Literature review requires a major rework. Address each reference individually not as a group without any comment on contents of each. 1] [2] [3] [4] [5] [6] [7] [8] [9] [10] [11] [12]; 20] [21] [22] [23] [24; [25] [26] [27] [2; ] [31] [32] [33] [34] [35] [36] [37] [38] [39] [40] [41] [42] [43] [44] [45] [46] [47] [48] [49] [5;. [66] [67] [68] [69] [70] [71] [72] [73] [74] [75] [76] [77] [78] [80] [81] [82] [83]; 101] [102] [103] [104] [105] [106] [107] [108] [109] [110] [111] [112] [113] [114]. A lot of the references seem to be inserted at random to inflate the overall number and does not bring much added value. Dedicated text must be added to each reference explaining results and relation to the paper.**

Response:

Thank you for your suggestion. We have added comments on each reference.

**2.　 Figure 3 should be a proper UML.**

Response:

We thank the reviewer for this comment. A new UML class diagram of our proposed system has been added as Figure 3. Therefore, the model diagram that was previously Figure 3 has been changed to Figure 4 which is a conceptual diagram to illustrate the layer structure of a complex model, so we have provided the simplest illustration.

**3. Still not all hyper parameters are clearly listed, nor reasoning exists on how they were determined. Some are in figure 3. I suggest also adding a dedicated table.**

Response:

Thank you for your comment. A new explanation of hyperparameters has been added as Figure 3. In addition, the followings were added to "3.1 CNN-LSTM model".

“The hyper-parameters of the LSTM were determined through grid search by comparing validation scores.”

“The hyper-parameters of these CNNs were determined through grid search by comparing validation scores. The mean and standard deviation parameters used in the standardization function were calculated from the entire image of training set.”

**4. Draw confusion matrix in color.**

Response:

Thank you for your comment. We have redrawn confusion matrix in color.

**5. Figure are low res and hard to see.**

Response:

Thank you for your comment. Figures 2, 4, and 5 have been changed to vector images. These have improved the resolution of the figures.

**6. Raw output data still not provided (data you draw all your results from)**

Response:

Thank you for your comment. All raw data has been redistributed at the following URL. This URL is provided in the introduction. In addition, programs for score calculation, t-SNE, and T-test are now available at this URL.

https://drive.google.com/drive/folders/1yqKXnDFhRCyUmO7x2-wFZG9oLnBiB8L1?usp=sharing

**Reviewer 2**

1. **The website:**

**https://github.com/ShotaroKataoka/work%20detection%20factory%20pretrain is not available**

Response:

Thank you for your comment. It appears that the PDF link-click feature was incompatible with the underbar in the URL. Therefore, the URL was modified to be clickable.
